# Versatility of the perforator radial artery flap in the reconstruction of the upper limbs and comparison of the outcomes with the “classic” radial flap, a retrospective study

**DOI:** 10.1016/j.jor.2023.08.006

**Published:** 2023-08-16

**Authors:** Camillo Fulchignoni, Giuseppe Rovere, Tommaso Greco, Carlo Perisano, Amarildo Smakaj, Andrea Fidanza, Lorenzo Rocchi, Elisabetta Pataia

**Affiliations:** a Hand Surgery and Orthopedics Unit, Department of Orthopaedics and Traumatology, Fondazione Policlinico Universitario A. Gemelli, IRCCS, Università Cattolica del Sacro Cuore, Rome, Italy; b Department of Orthopaedics and Traumatology, Fondazione Policlinico Universitario A. Gemelli, IRCCS, Università Cattolica del Sacro Cuore, Rome, Italy; c Department of Life Health & Environmental Sciences, University of L'Aquila, Mininvasive Orthopaedic Surgery, L'Aquila, Italy

**Keywords:** Radial artery flap, Chinese flap, Perforators, Cardiovascular issues, Versatility

## Abstract

**Introduction:**

Radial forearm flap, first described in the early eighties in China, is a well-known and handy flap to cover soft tissue defects of the distal upper limb. It has, though, some inconveniences, such as the sacrifice of the radial artery and non-neglectable esthetic sequelae in the donor site. In the following years, a similar flap based on the perforators of the radial artery has been described as achieving similar results, allowing to spare a main vessel. The authors reviewed retrospectively the patients that underwent surgery with one of those two flaps in their center to compare outcomes.

**Materials and methods:**

Patients operated between January 2016 and January 2022 have been reviewed. Ten had a classic radial artery flap, and ten had a radial artery perforator flap. Twelve weeks after surgery, Vancouver Scar Scale was used to assess the results at the donor site and over the flap. Reintervention and failure rate within one year and patient satisfaction -using a visual analog scale ranging from 0 to ten-at 12 months were also assessed.

**Results:**

All classic radial artery flap group patients had “successful” surgery, and none needed secondary surgery. On the other side, three patients required a second surgery in the perforator flap group, and nine out of ten ended up with “successful” flaps. Mean Vancouver Scar Scale results regarding the flap are comparable, whereas those at the donor site are significantly better in the patients with the perforator flap. Patients’ satisfaction results are similar in both groups.

**Conclusion:**

The radial artery perforator flap is an important flap to be held in mind by all surgeons approaching reconstruction of the elbow, the forearm, and the hand, and should be preferred, when possible, to the classic radial forearm flap.

## 1 Introduction

Coverage of soft tissue loss represents an essential part of any hand surgery department activity. Providing good soft tissue coverage necessary to protect vital structures, achieving an early and complete functional outcome, and obtaining an esthetic result as good as possible are among the principal objectives in the reconstruction of soft tissue defects in the hand and the forearm.^1^^,^^2^ To treat those kinds of defects, recent advances regarding the biological repair and skin blood supply research, and increased knowledge in reconstructive surgery, a continuously growing number of techniques are available for the hand surgeon.

Although the skin of the palmar region and dorsal region of the hand differs in thickness and flexibility, the radial forearm flap (RFF) represents a good option in both cases. This flap was first described in the early eighties in China^3^^,^^4^ and then rapidly diffused in Europe, where it became known as the “Chinese flap”.^5^ As indicated by its name, this flap foresees the sacrifice of an important vessel, such as the radial artery, allowing surgeons to dissect a very large flap up to 25 cm × 15 cm.^6^ The flap has been described as pedicled with retrograde flow to cover soft tissue defects of the hand – including fingers, thumb, first commissure, dorsal and palmar region^7^^,^^8^- or with an anterograde flow to cover soft tissue defect of the elbow region.^9^ Thanks to its possible wide design, it can also be used as a free flap for as many different applications as imaginable such as esophageal reconstruction^10^ or phalloplasty.^11^ Furthermore, this flap can be used as a composite, including skin, fascia, tendons, and/or bone. Advantages of this flap include good reliability, the presence of a long and large pedicle, the possibility to elevate a wide flap, and to include other tissues. The main disadvantages are represented by the sacrifice of a main artery of the forearm – if the Allen test^12^ is negative, this flap cannot be used- and non-neglectable esthetic sequelae in the donor site.^13^

At the end of the same decade, with the increased knowledge of perforator vessels and the definition of “angiosomes” by Taylor,^14^ surgeons started to harvest their flaps on those perforators. At the forearm level, the first cases were published in 1988,^15^ allowing to spare the radial artery. The radial artery perforator flap (RAPF) can reach up to 20 × 10 cm in width. It can be harvested as a fascio-cutaneous flap or as an adipo-fascial flap with a proximal pedicle (inferior cubital artery) or a distal pedicle (palmar branch of the radial artery at 2–4 cm proximally to the radial styloid).^16^ The proximal pedicle variant of the RAPF is rarely used because of the existence of local flaps that can cover the elbow region. Some surgeons use this perforator flap also as a free flap.^17^ The advantages of the RFAP are the sparing of a major vessel, lower donor site morbidity, and constant presence and anatomy of perforators. Disadvantages include a smaller dimension of the flap, a shorter pedicle (RAPF can cover hand tissue loss up to proximal phalanges of the hand), and a higher rate of complications due to the more delicate nature of perforators.^18^

To the authors' knowledge, there is no study, up to now, in the literature comparing outcomes of the RAPF and the classic RFF. Therefore, the authors decided to review retrospectively patients treated in their center with one of those two flaps for reconstructions of the elbow, forearm, and hand soft tissue defects.

## 2 Materials and methods

This is a retrospective study approved by the local ethical committee, including patients operated between January 2016 and January 2022 in the authors' department. To be included in the study, cases had to meet the following inclusion criteria: a tissue loss of a minimum of 5 cm × 5 cm in the elbow, forearm, or hand treated either with a classical RFF or a RAPF; age ranging between 18 and 70 years old; minimum follow-up of one year. Exclusion criteria included: contemporary bone synthesis and flaps that did not include skin (for easier assessment of flap success or failure).

Twenty patients were included in this study: ten have been treated with a classic radial forearm flap (group A) and ten with a radial artery perforator flap (group B). All patients of group A had a positive Allen test, and all patients of group B had a Doppler examination to locate the perforator before surgery.

All procedures were performed by the same experienced surgeon (E.P.) following written informed patient consent and in accordance with the ethical standards of the institutional and/or national research committee and the 1964 Declaration of Helsinki and its subsequent amendments or comparable ethical standards.

Patients were not immobilized and were dismissed from the hospital two days after surgery. Patients were then checked for follow-up at 1, 2, 4, 8, and 12 weeks and then at 6 and 12 months. Twelve weeks after surgery, Vancouver Scar Scale (VSS)^19^ was used to assess the results at the donor site and over the flap. Reintervention and failure (need to remove the flap) rate within one year and patient satisfaction -using a visual analog scale (satiVAS) ranging from 0 to ten-at 12 months were also assessed.

Continuous variables (age, VSS, and satiVAS) are presented as means, range, and standard deviation. The significance of differences between two means is assessed using the t-statistic calculated as part of the two-tailed t-student test with a confidence level alfa = 0,05. The significance of the evolution of bounded scores (reintervention and failure rates) is assessed by comparing their distributions using a two-sided chi-square test with a confidence level alfa = 0,05.

## 3 Results

### 3.1 Patients, etiology of tissue loss, and characteristics of flaps

Among the twenty patients in this study, eleven were male, and nine were female. The mean age was 53 years old (details in Table 1).

**Table 1:** Patients.

		Group A	Group B	Overall
**Mean age (in years)**		51	55	53
Range		(23–68)	(35–69)	(23–69)
Standard Deviation		12.5	10.9	12.2
**Sex**	*Male*	5	6	11
	*Female*	5	4	9

In group A, six patients had a loss of tissue consequent to a traumatic event, three due to the excision of a tumoral mass, and one had a vasculitic ulcer. The ulcer patient had a loss of tissue at the elbow and was treated with an RFF with a proximal pedicle, whereas the nine other patients had a loss of tissue at the wrist and hand level and were treated with an RFF distally pedicled.

In group B, loss of soft tissue was consequent to a traumatic event in four patients, two cases were oncological, two patients had a vasculitic ulcer, one had an ulcer due to radiodermatitis, and one patient had a loss of tissue of the dorsum of the hand following an S. Aureus infection. In this group, two other patients had a loss of tissue at the dorsum of the hand (a degloving injury and a patient with sarcoma). Two patients needed a flap to reconstruct the first web space (a blast trauma and a vasculitic ulcer), and three patients to reconstruct the distal forearm (one sarcoma and two crush trauma). Those eight patients with tissue loss at the forearm or the hand level were treated with a RAPF with a distal pedicle. Two patients with soft tissue loss at the elbow (vasculitic ulcer and ulcer due to radiodermatitis) were treated with RAPF with a proximal pedicle.

### 3.2 Objective and subjective outcomes

All group A patients had “successful” surgery; no patients needed secondary surgery. In group B, three patients required a second surgery (see complication paragraph below), and nine ended up with “successful” flaps. Mean VSS regarding the flap is comparable, whereas results for the VSS at the donor site are significantly better in the patients of group B. Finally, patients’ satisfaction results are similar in both groups. Complete results and p values are reported in Table 2.

**Table 2:** Results.

	Group A	Group B	P value
**Success rate (%)**	100	90	0.00118
**Secondary surgery rate (%)**	0	30	<0.00001
**Mean VSS flap**	3.9	3.75	0,80,141
Range	(2–7)	(2–5)	/
Standard Deviation	1.4	1.0	/
**Mean VSS donor site**	6.1	3.1	0.00002
Range	(3–8)	(2–5)	/
Standard Deviation	1.7	1.0	/
**satiVAS**	8.9	9.2	0.43319
Range	(7–10)	(8–10)	/
Standard Deviation	1.0	0.6	/

### 3.3 Complications

No complications were reported in group A patients. On the other side, three patients in group B had complications that led to revision surgery. In two cases, the superficial cutaneous layer of the flap suffered while the underlying tissue was vital. Therefore, those patients underwent skin excision followed in one case by a skin graft, whereas, in the other, the patient healed by secondary intention healing. Both those patients had cardiovascular issues: one had a bypass four years before RAPF, whereas the other had a positive history of upper limb thrombosis. The third patient who underwent a second surgery is a patient with a sarcoma of the forearm, which had chemo- and radiotherapy before surgery. In this case, the whole flap was necrotic and therefore excised. The patient was treated with a dermal substitute until the end of her adjuvant therapy, and then reconstruction was achieved with an anterolateral thigh flap.

### 3.4 Clinical cases

To better illustrate the versatility of the RAPF, the authors of this study will present two clinical cases.

**Fig. 1:**
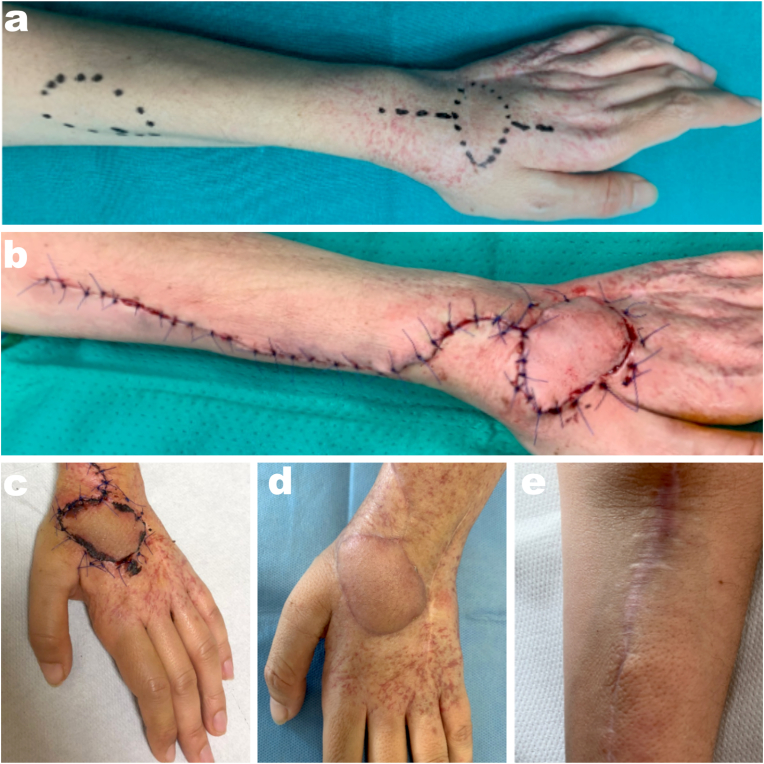
a patient with a synovial sarcoma recurrence at the dorsum of the left hand, needing an RFAP distally pedicled to cover the oncological excision. (a) preop drawing; (b)immediate postop; (c) 2 weeks follow-up; flap (d) and donor site (e) at one-year follow-up.

Case One is a 55 years old female (Fig. 1) who came to the authors' attention for a synovial sarcoma recurrence at the dorsum of the left hand. She previously (three years before) underwent excision of the sarcoma and closure by direct suture of the skin, followed by radiotherapy, in another center. When reaching the author's center, the patient also suffered from radiodermatitis. After the MRI study, excision -including the skin-was planned. The authors harvested an adipo-fascio-cutaneous RAPF of 6 cm × 5 cm with a distal pedicle to reconstruct the soft-tissue defect. The donor site was closed by direct suture. At the one-year follow-up patient had excellent results in terms of VSS, both for the donor site and the flap, and in terms of patient satisfaction.

**Fig. 2:**
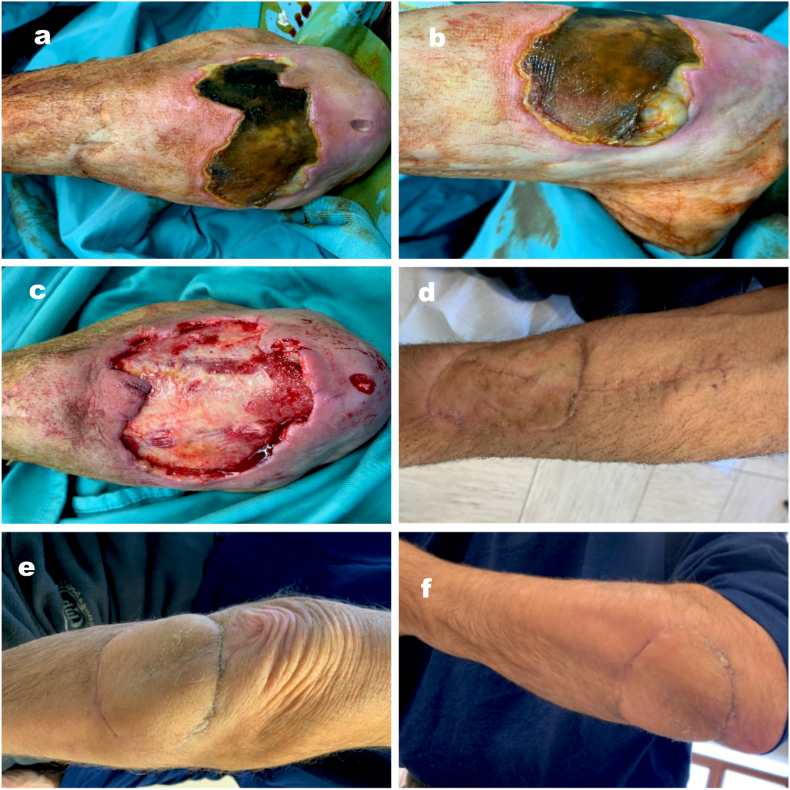
a patient with a cutaneous necrotic ulcer due to vasculitis, needing an RFAP proximally pedicled to cover the eschar excision. (a–b) immediate preop; (c) intraop, after eschar excision; donor site (d) and flap (e–f) at one-year follow-up.

Case two is a 64 years old male (Fig. 2) who was first seen by the authors when hospitalized in the Rheumatology ward for a cutaneous necrotic ulcer due to vasculitis in a patient with rheumatoid arthritis. The patient underwent an eschar excision that exposed the bone and was reconstructed with a fascio-cutaneous RAPF of 6 cm × 8 cm proximally pedicled. The donor site was covered with a thin skin graft. The patient achieved excellent results in terms of satisfaction and flap quality and good results at the donor site.

## 4 Discussion

Radial artery perforator flap has been described over 30 years ago^15^ and demonstrated to be a valuable tool for achieving good results in upper limb reconstruction. Nevertheless, few articles and very few series are available in the literature.

In this study, the authors have demonstrated how RAPF can be helpful in covering different types of defects of various sizes and numerous localizations of the distal upper limb. Furthermore, the authors achieved results comparable, if not better, to those of RFF regarding scar quality at the flap and the donor site level, and patient satisfaction. The success rate was also very high, with only one failure in this series. RAPF seems to be a very safe flap, as results reported in the literature are comparable to those of this study. Numerous authors^18^^,^^20^, ^21^, ^22^ reported no failures in reconstruction achieved with RAPF. The same authors agree that functional outcomes depend mainly on the characteristics (tissue involved, size, localization) of the initial tissue loss rather than on which flap is used to reconstruct it.

Regarding complications, the most commonly described in the literature is partial flap necrosis. In Jeng et al. series,^21^ one flap in one patient out of twelve had a distal tip necrosis that healed by second intention. Chang et al.^22^ described two patients (out of 34) who needed revision surgery for minor flap loss (<10%). More recently, Matei et al.,^18^ in their article about all perforator flaps of the forearm, presented 44 patients receiving a RAPF. Two patients had partial flap necrosis, needing a skin graft to heal. Overall, among all the perforator flaps in their study, patients had epidermolysis in 12% of cases due to transitory venous congestion. Similarly, in this study, authors describe two cases of partial flap necrosis. Those complications, rarer with the RFF, are ascribable to the fragility of smaller vessels of the RAPF.^16^ Furthermore, in this study, patients who had complications were all dealing with cardiovascular issues, confirming indications found in the literature that the use of this flap should be limited to patients without any microvascular arterial disease risk, including diabetics and smokers, among others.^16^

In this article, the authors included cases of RAPF, with both proximal and distal pedicles, to illustrate the versatility of those flaps. Indications for a proximally pedicled flap, also used by other authors,^23^ are more limited due to the existence of other local and regional flaps available in this region.^24^^,^^25^ On the other hand, distally pedicled flaps are very useful and can cover different areas of the distal upper limb. In this series, similarly to the other series available in the literature,^18^^,^^20^, ^21^, ^22^ distally pedicled RAPF is used to cover defects of the distal forearm, the dorsum of the hand, the thumb, and of the first web space. Some authors also use it for the volar region of the hand.^21^^,^^22^ Given its great versatility, RAPF has also been described for other pathologies, such as treatment for radio-ulnar synostosis.^26^ Finally, when used as a free flap, RAPF can be used to cover any region of the body, for example, distal finger tissue losses.^27^

This study presents numerous limitations, such as its retrospective aspect, the small number of patients included, the inclusion of both proximally and distally pedicle flaps (which might be considered different flaps by some authors), and the heterogeneity of loss of tissue etiologies. Despite those limitations, the authors believe that they can conclude that the radial artery perforator flap is an important flap to be held in mind by all surgeons approaching reconstruction of the elbow, forearm, and hand.

## 5 Conclusion

Relying on the results of this study, authors believe that when possible, the RAPF should be preferred to the classic radial forearm flap, along with other perforator flaps of the region.^28^^,^^29^ This flap allows to cover tissue losses of big sizes and of numerous anatomical areas, achieving excellent esthetic results at the donor and receiving site, sparing main vessels. Nevertheless, in patients with cardiovascular issues, diabetics, or smokers, the classic radial forearm flap might be more appropriate to avoid complications.

## Funding

This research received no external funding.

## Informed consent statement

Informed consent to treat data was obtained from all subjects involved in the study. The study is in accordance with the ethical standards of the 1964 Helsinki declaration and its late amendments.

## Data availability statement

Data is available on demand, asking the corresponding author.

## Authors contribution

Conceptualization, CF, EP and LR; methodology, CF and GR; validation, EP, LR and CP; formal analysis, CF; resources, CF and EP; data curation, GR, TG, AF and AS; writing—original draft preparation, CF, GR and CP; writing—review and editing, CF, AF and AS; visualization, TG; supervision CP, LR and EP; all authors have read and agreed to the published version of the manuscript.

## Use of AI tool

No AI tool has been used.

## Declaration of competing interest

The authors declare no conflict of interest.
